# Sheet-Like Morphology CuO/Co_3_O_4_ Nanocomposites for Enhanced Catalysis in Hydrogenation of CO_2_ to Methanol

**DOI:** 10.3390/nano13243153

**Published:** 2023-12-16

**Authors:** Zhenteng Sheng, Hui Zhou, Yuhua Zhang, Jinlin Li, Li Wang

**Affiliations:** Key Laboratory of Catalysis and Energy Materials Chemistry of Ministry of Education, Hubei Key Laboratory of Catalysis and Materials Science, South-Central Minzu University, Wuhan 430074, China

**Keywords:** CO_2_ hydrogenation, methanol, cobalt, copper

## Abstract

The selective hydrogenation of CO_2_ into high-value chemicals is an effective approach to address environmental issues. Cobalt-based catalysts have significant potential in CO_2_ hydrogenation reaction systems; however, there is a need to control their selectivity better. In this study, copper is introduced onto Co_3_O_4_ nanosheets using the ion exchange reverse loading method. The unique interaction of these materials significantly alters the selectivity of the cobalt-based catalyst. Results from scanning transmission electron microscopy and scanning electron microscopy indicate that this catalyst enables a more even dispersion of copper species in the Co_3_O_4_ nanosheets. Temperature-programmed reduction and X-ray photoelectron spectroscopy reveal that the catalyst facilitates the metal–metal interaction between Co and Cu. Temperature-programmed desorption experiments for CO_2_ and H_2_ demonstrate that the close interaction between Co and Cu modifies CO_2_ adsorption, leading to differences in catalytic activity. Moreover, the catalyst effectively suppresses CO_2_ methanation and promotes methanol formation by altering the alkalinity of the catalyst surface and weakening the hydrogen dissociation ability.

## 1. Introduction

The environmental problem caused by substantial CO_2_ emissions has gained prominence, and an effective way to solve this problem is to pursue a low-carbon, energy-efficient CO_2_ treatment method to reduce CO_2_ emissions [1]. Renewable hydrogen, generated from water using clean electricity, can be used to convert CO_2_ into valuable chemicals like methane, syngas, long-chain hydrocarbons, olefins, alcohols, and more. This approach offers an effective strategy to reduce carbon emissions and promote the utilization of carbon resources [2,3]. The inherent chemical inertness of CO_2_ often presents a substantial energy barrier in reactions. Consequently, the direct conversion of CO_2_ into valuable chemicals remains a challenging task due to the intricate reaction pathways and the diversity of intermediates involved [4,5,6].

Methanol, as an essential chemical feedstock, serves as an excellent hydrogen storage material and fuel. It can be utilized in the production of a wide range of high-value-added chemicals [7,8]. Catalysts for CO_2_ hydrogenation to methanol encompass a variety of options, including copper-based catalysts [9], indium-based catalysts [10], noble metal catalysts [11], Zn-Zr solid solutions [12], and cobalt-based catalysts [13]. The CO_2_ hydrogenation pathway involves the reverse water–gas shift (RWGS) reaction between CO_2_ and hydrogen to produce CO, which is further hydrogenated to form methanol [14,15]. Thus, the catalysts designed for CO_2_ hydrogenation to methanol are often modified to facilitate CO hydrogenation to methanol. Copper-based catalysts, owing to their weak adsorption of H atoms, exhibit excellent RWGS reaction activity and nearly 100% selectivity for CO [16]. Consequently, the addition of Cu as a promoter to enhance the formation of a crucial intermediate species during CO_2_ hydrogenation to methanol is an effective approach for boosting methanol selectivity.

Cobalt-based catalysts have been widely used in the Fischer–Tropsch synthesis industry, primarily because of their impressive ability to cleave C-O bonds and dissociate H_2_ [17,18]. However, the primary product obtained in CO_2_ hydrogenation over the cobalt-based catalyst is methane [19]. This is primarily attributed to the electronic configuration of the metal Co that promotes the disruption of hydrogen bonds, leading to the formation of active hydrogen species, favoring its hydrogenation properties [20]. Single-component, cobalt-based catalysts predominantly yield products such as CH_4_ and CO, irrespective of whether cobalt is present as Co_3_O_4_, CoO, or Co^0^ [21]. Previous studies have demonstrated that CoO and Co^0^ can each facilitate the conversion of CO_2_ into either CO or CH_4_, indicating that CoO tends to favor RWGS reaction; meanwhile, Co^0^ tends to favor methanation. Additionally, the selectivity of cobalt-based catalysts can be significantly altered by manipulating the redox properties of cobalt sites or modifying the coordination environment [13,22,23,24,25]. In the study conducted by Xiao et al. on the Co@Si_0.95_ catalyst [13], it was revealed that the presence of Co-O-SiO_x_ played a stabilizing role in maintaining the oxidation state of cobalt within the catalyst. This slowed down the cleavage of the C-O bond in the critical intermediate CHO_3_*, ultimately leading to the formation of methanol. Gasconetal et al. reported that the Co-In catalyst improves methanol selectivity by promoting interactions between Co-CoO_x_ and In_2_O_3_ within this core–shell structure [26]. Wu et al. reported that phosphating Co-Al-LDH (double-layered hydroxide) was prepared, and they found a clear electron transfer in the oxygen vacancies on the surface of the catalyst. This electronic effect inhibits the cleavage of CH_3_*O C-O bonds and increases the yield of methanol [27].

In combination, the robust capability of Co^0^ to cleave C-O bonds and dissociate H_2_ can be finetuned by introducing a second component, thereby enhancing the interaction between cobalt and the additional metal. This modification serves to fine-tune the reaction pathway of cobalt-based catalysts, facilitating the product formation toward CO while suppressing methanation. Cu-based catalysts are extensively employed in the industrial production of methanol via CO_2_/CO hydrogenation. The simultaneous utilization of CuO as a photocatalyst for the conversion of CO_2_ to methanol holds great promise in the field [28]. Currently, Co-Cu bimetallic catalysts find extensive applications in catalytic reactions such as synthesis gas conversion to higher alcohols [29], toluene degradation [30], and furfural hydrogenation [31]. However, further investigation is still required for the application of CO_2_ hydrogenation in catalytic reactions. The primary focus of reported Co-Cu catalysts for CO_2_ hydrogenation lies in the construction of a Co-Cu alloy; yet, there still exist certain challenges, such as the limited product yield. Ion exchange represents a novel approach to catalyst preparation [32]. Conventional catalyst preparation methods [33], such as impregnation and precipitation, often encounter issues related to a limited contact area/distance between the active center and the support, as well as weak interfacial interactions. The ion exchange method can modify the coordination environment of the original components and establish metal–metal interactions through lattice insertion.

Herein, an ion exchange method was utilized to incorporate copper into Co_3_O_4_ nanosheets, aiming to enhance the interaction between Co and Cu. Compared to the impregnation method, this approach resulted in a significant improvement in the selectivity for methanol in the CO_2_ hydrogenation process. The primary factors contributing to this improvement include the Co-Cu bimetallic interaction, surface coordination morphology, and spatial arrangement.

## 2. Experimental Procedures

### 2.1. Chemicals

Co(OH)_2_ was purchased from Aladdin; Cu(NO_3_)_2_·3H_2_O and C_2_H_5_OH from Sinopharm. All chemicals were of high purity (>99.0%) and used as received without additional purification.

### 2.2. Synthetic Procedures

#### 2.2.1. Synthesis of CuO/Co_3_O_4_-IE

Briefly, 0.74 g Cu(NO_3_)_2_·3H_2_O was dissolved in 50 mL distilled water, followed by adding 1.2 g Co(OH)_2_ to the Cu(NO_3_)_2_·3H_2_O aqueous solution and stirring for 30 min. Next, the dispersion was transferred into a 100 mL Teflon-lined stainless steel autoclave and heated at 120 °C for 12 h. After cooling to room temperature, the sample was centrifuged and washed three times in distilled water. Subsequently, the obtained sample was dried in an oven at 80 °C for 6 h. After cooling to room temperature, the sample was ground to a powder with an agate mortar and then calcined in a muffle furnace at 350 °C for 3 h. The resulting black powder sample was named CuO/Co_3_O_4_-IE.

#### 2.2.2. Synthesis of CuO/Co_3_O_4_-IM

Briefly, 0.74 g Cu(NO_3_)_2_·3H_2_O was dissolved in 50 mL ethanol, followed by adding 1.2 g Co(OH)_2_ to the Cu(NO_3_)_2_·3H_2_O aqueous solution and ultrasonic stirring for 30 min. The mixed solution was added to the rotary evaporator, and the flask pressure was lowered to 0.1 MPa using a vacuum pump. The temperature of the rotary evaporator was then set to 50 °C for drying, followed by further treatment in an oven at 80 °C for 6 h. After cooling to room temperature, the sample was ground to powder with an agate mortar and then calcined in a muffle furnace at 350 °C for 3 h. The resulting black powder sample was named CuO/Co_3_O_4_-IM.

#### 2.2.3. Synthesis of Co_3_O_4_

The black powdered Co_3_O_4_ was obtained by calcining 2 g of Co(OH)_2_ in a muffle furnace at 350 °C for 3 h.

### 2.3. Characterization

X-ray diffraction (XRD) patterns were obtained using a Bruker D8 powder diffractometer (Karlsruhe, Germany) equipped with a Cu-K_α_ radiation source operated at 40 kV and 40 mA along with a Vantec-1 detector. The size and morphology of the samples were determined using an FEI Tecnai G20 transmission electron microscope operated at 200 kV and a Hitachi SU8000 field emission scanning electron microscope operating at an accelerating voltage of 15 kV. X-ray photoelectron spectra (XPS) analyses were performed on a VG Multilab 2000 spectrometer (New York, NY, USA), using Al-K_α_ radiation. Temperature-programmed desorption (TPD) and temperature-programmed reduction (TPR) experiments were conducted on a Zeton Altamira AMI-300 (Beijing, China). The concentrations of Co and Cu ions were examined using inductively coupled plasma mass spectrometry (ICP-MS, Agilent 7700s, Santa Clara, CA, USA).

### 2.4. Catalytic Testing

The CO_2_ hydrogenation reaction was performed in a fixed-bed reactor equipped with a stainless-steel tube with an inner diameter of 1/4 in. The catalyst was reduced at 150 °C using pure H_2_ (4 L·g_cat_^−1^·h^−1^) for 1 h, followed by cooling to 50 °C in flowing H_2_ before introducing the feed gas (H_2_/CO_2_/N_2_ = 22.5%/67.5%/10%, with N_2_ as the internal standard). The gas hourly space velocity (GHSV) was maintained as 6 L·g_cat_^−1^·h^−1^, while the pressure was set at 2.0 MPa, followed by increasing the reaction temperature slowly to 250 °C. During the reaction, the effluent gases from the reactor (H_2_, CO, CO_2_) and light alkanes were continuously monitored using an online gas chromatograph (Agilent GC 7890B, Santa Clara, CA, USA). The liquid products were condensed in a cold trap (273 K) and analyzed using an Agilent 4890 GC (Santa Clara, CA, USA) equipped with a flame ionization detector (FID) and an HP-Innowax column. The product selectivity was calculated based on the carbon balance.

The CO_2_ conversion and selectivity were defined using Equations (1) and (2), respectively:

(1) $$X_{{\mathrm{CO}}_{2}}=\frac{{\mathrm{CO}}_{2,\mathrm{in}}\left(\mathrm{mol}\right)-{\mathrm{CO}}_{2,\mathrm{out}}\left(\mathrm{mol}\right)}{{\mathrm{CO}}_{2,\mathrm{in}}(\mathrm{mol})}$$

(2) $$\mathrm{Selectivity}=\frac{n_{\mathrm{product},\mathrm{out}}(\mathrm{mol})\times n}{n_{{\mathrm{CO}}_{2,\mathrm{in}}}(\mathrm{mol})-n_{{\mathrm{CO}}_{2,\mathrm{out}}}(\mathrm{mol})}$$

where CO_2,in_ and CO_2,out_ represent the inlet and outlet molar flow rates of CO_2_, respectively; n_product,out_ is the outlet molar flow rate of the specific product, and n represents the stoichiometric coefficient of CO_2_ in the balanced reaction equation.

## 3. Results

### 3.1. SEM and STEM

**Figure 1:**
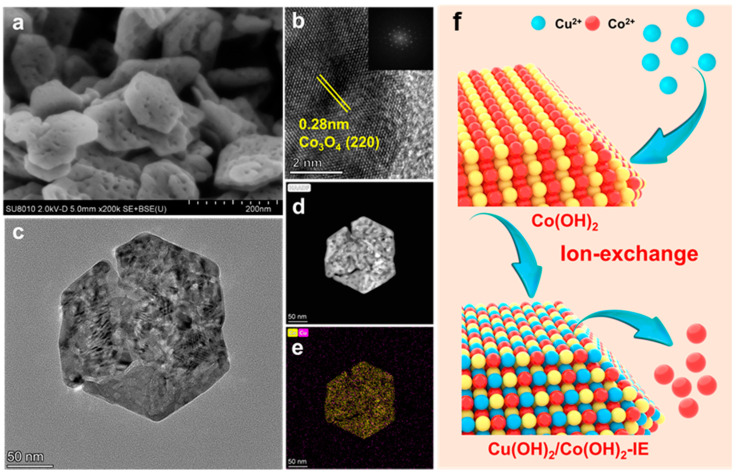
(**a**) SEM image, (**b**) HR-STEM image, and (**c**–**e**) STEM image, HAADF-STEM image, and EDX elemental mappings of CuO/Co_3_O_4_-IE, with yellow for Co and purple for Cu; (**f**) illustration of the preparation of the Cu(OH)_2_/Co(OH)_2_-IE nanocomposites.

The morphology and element distribution of the CuO/Co_3_O_4_-IE catalyst were analyzed using scanning electron microscopy (SEM) and scanning transmission electron microscopy (STEM). After calcination, the catalyst was seen to maintain the shape of a Co(OH)_2_ hexagonal nanosheet (Figure 1a and Figure S1), exhibiting a tiny part broken due to calcination. Additionally, due to the dehydration of hydroxide caused by high-temperature calcination, there are many disordered porous structures (Figure 1c) on the nanosheets. High-angle annular dark-field scanning transmission electron microscopy (HAADF-STEM, Figure 1d) and EDX elemental mappings (Figure 1e) show the uniform dispersion of cobalt and copper, with no significant copper oxide nanoparticles observed in the results of either SEM or STEM. In the STEM images (Figures S2 and S3), it is shown that compared with the catalyst (CuO/Co_3_O_4_-IE) prepared using the ion exchange method (Figure S3), the flake structure of the catalyst (CuO/Co_3_O_4_-IM) prepared using the impregnation method is significantly destroyed (Figure S2) after calcination. At the same time, a partial clustering of cuprates can be seen in the EDX elemental mappings. SEM and STEM images show that the catalysts prepared using the two methods can effectively disperse copper species; however, the ion exchange method can achieve closer contact between the two components. The schematic diagram of catalyst synthesis is shown in Figure 1f.

### 3.2. Diffraction of X-rays (XRD) and ICP

**Figure 2:**
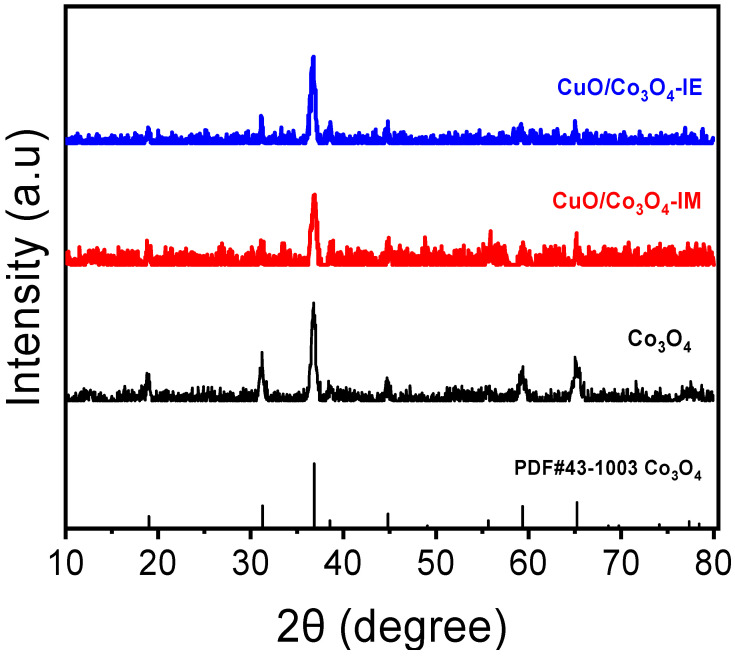
XRD patterns of CuO/Co_3_O_4_-IE, CuO/Co_3_O_4_-IM, and Co_3_O_4_ catalysts after calcination.

The XRD patterns of CuO/Co_3_O_4_-IE, CuO/Co_3_O_4_-IM, and Co_3_O_4_ catalysts are shown in Figure 2. After calcination at a high temperature, the characteristic diffraction peaks of Co_3_O_4_ (PDF#43-1003) are shown in the three catalysts, indicating the existence of Co in the form of the Co_3_O_4_ phase in them. However, the characteristic diffraction peaks associated with copper species were not observed in either of the catalysts. Based on comprehensive scanning transmission electron microscopy (STEM) and energy-dispersive EDX elemental mappings characterization, it is postulated that copper exists in a highly dispersed amorphous CuO state within the catalyst, thereby accounting for this phenomenon. The ICP-MS characterization results for the CuO/Co_3_O_4_-IE and CuO/Co_3_O_4_-IM catalysts are presented in Table 1. The molar ratio of cobalt to copper in both catalysts is approximately equal.

### 3.3. Surface Chemical Analysis and Chemical Adsorption

**Figure 3:**
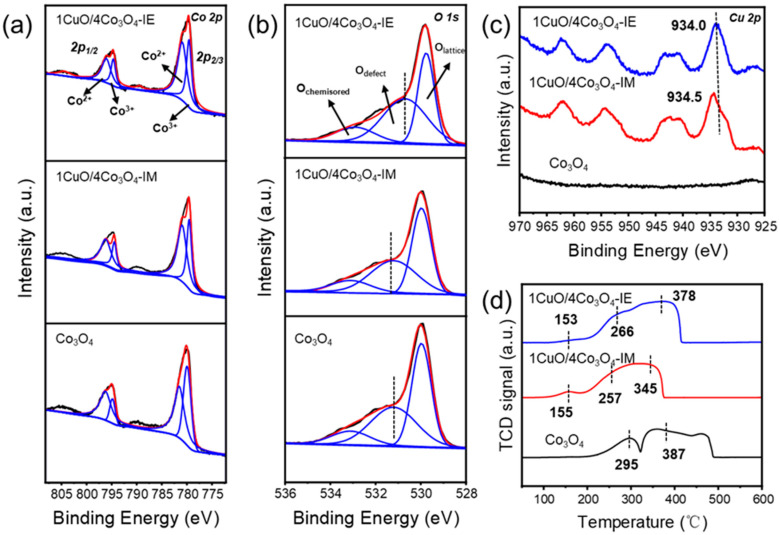
XPS spectra for CuO/Co_3_O_4_-IE, CuO/Co_3_O_4_-IM, and Co_3_O_4_: (**a**) Co 2p, (**b**) O 1s, (**c**) Cu 2p; (**d**) The H_2_-TPR profiles of CuO/Co_3_O_4_-IE, CuO/Co_3_O_4_-IM, and Co_3_O_4_.

The surface chemical state of the Co, Cu, and O elements in CuO/Co_3_O_4_-IE, CuO/Co_3_O_4_-IM, and Co_3_O_4_ catalysts was studied using X-ray photoelectron spectroscopy (XPS). Figure 3a shows the Co 2p spectra of cobalt in all the catalysts; moreover, by peak separation fits, all catalysts are seen to show typical asymmetric Co 2p_3/2_, Co 2p_1/2_, and satellite peaks at binding energies of 780.1, 786.3, 797.3, and 805.2 eV, respectively. The two peaks around 780.0 and 795.5 eV are associated with Co^3+^, and the two peaks around 781.6 and 797.1 eV belong to Co^2+^, indicating the coexistence of Co^2+^ and Co^3+^ in the catalyst. These observations are consistent with the XRD results, indicating the presence of Co in the form of Co_3_O_4_ in the three catalysts [34]. In Table S1, the binding energy of Co is seen to shift relative to the binding energies of Co_3_O_4_, CuO/Co_3_O_4_-IE, and CuO/Co_3_O_4_-IM on the Co 2p_2/3_ orbitals, indicating that the incorporation of Cu changes the coordination environment and electronic state around Co, leading to a metal-to-metal interaction [31,35]. In the Cu 2p spectra of all catalysts (Figure 3c), the correlation peak of Cu is not observed in the Co_3_O_4_ catalyst, and the Cu 2p orbital peak is shifted for the catalyst prepared using the ion exchange method relative to the catalyst prepared using the impregnation method. Combined with the Cu 2p orbital spectra, the interaction and electron transfer can be inferred to be stronger for Co-Cu prepared using the ion exchange method. The O 1s spectra of oxygen elements for all catalysts are shown in Figure 3b, and the three peaks at about 530.1, 531.4, and 533.2 eV correspond to lattice oxygen (O_lattice_), defect oxygen (O_defect_), and chemisorbed oxygen (O_chemisored_), respectively [36]. The density of defect oxygen (O_defect_) in the catalyst was evaluated by integrating the peak area (P) of O_lattice_ and O_defect_ O_chemisored_, (O_defect_ = PO_defect_/(PO_defect_ + PO_lattice_ + PO_chemisored_) × 100%) [37]. An analysis of all the catalysts revealed that the concentration of oxygen vacancies on their surface definitely decreases after the addition of Cu, which may also be responsible for the reduced catalyst activity (Table S2). However, there is a slight difference in oxygen vacancy between the two catalysts containing Cu. As shown in Figure 3b and Table S3, the binding energy of oxygen vacancies on the catalyst surface changes significantly after the introduction of copper via ion exchange, with oxygen vacancies having shifted to lower binding energies, in contrast to the absence of any obvious change in the oxygen vacancy on the catalyst surface for the introduction of copper using the impregnation method. This indicates that the catalyst introduced using the ion exchange method has a greater influence on the chemical properties of the catalyst surface, with a significant electron transfer occurring on the surface. A recent study has shown that electron transfer from oxygen vacancies can constrain the adsorption of CO_2_ and the activation of H_2_, thus inducing changes in the CO_2_ hydrogenation reaction pathway and increasing the selectivity of methanol [27], which may explain the change in the product selectivity of the CuO/Co_3_O_4_-IE catalyst in CO_2_ hydrogenation reactions. According to the surface elemental concentrations (Table S4) and ICP-MS characterization results (Table 1), while the Co and Cu content are similar, the Cu content on the surface of the CuO/Co_3_O_4_-IM catalyst notably surpassed that of the CuO/Co_3_O_4_-IE catalyst. This suggests that, in the CuO/Co_3_O_4_-IM catalyst, copper primarily resides on the surface of Co_3_O_4_ nanosheets. Conversely, in the CuO/Co_3_O_4_-IE catalyst, copper not only occupies the surface of Co_3_O_4_ nanosheets but also exists within the bulk phase via the ion exchange process.

The H_2_-TPR experiment was performed to illustrate the Co-Cu interaction. The H_2_-TPR reduction curve of Co_3_O_4_-based catalysts can be divided into three different temperature regions for discussion (Figure 3d). The reduction peak at 150 °C–200 °C belongs to the reduction in CuO→Cu (CuO/Co_3_O_4_-IE, CuO/Co_3_O_4_-IM); moreover, the reduction peak of single CuO appears at about 260 °C [38], and, in the Co-Cu two-component catalyst, the reduction peaks of CuO and Co_3_O_4_ tend to merge. The reduction peak at 200 °C–300 °C belongs to a reduction in Co_3_O_4_→CoO (all catalysts), while the reduction peak at 350 °C–500 °C belongs to a reduction in CoO→Co (all catalysts). These observations clearly indicate that the Cu addition promotes a reduction in the Co_3_O_4_ catalyst, especially a reduction in CoO→Co. Therefore, the reduction peak shift is attributed to the addition of Cu, leading to a certain interaction between the two components of Co-Cu, which is consistent with the XPS results.

**Figure 4:**
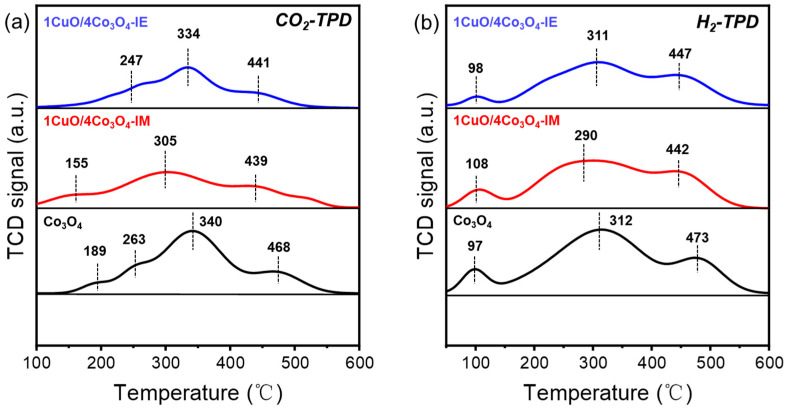
(**a**) CO_2_-TPD and (**b**) H_2_-TPD profiles of the CuO/Co_3_O_4_-IE, CuO/Co_3_O_4_-IM, and Co_3_O_4_ catalysts.

Figure 4 shows the CO_2_ and H_2_ temperature programmed desorption (TPD) diagrams of the three catalysts, and the gas desorption amounts of all catalysts are presented in Table S5. In Figure 4a, the addition of Cu is seen to reduce the amount of CO_2_ desorption in the middle- and low-temperature segments, resulting in a decrease in the number of surface alkaline sites, which may be responsible for the decrease in the catalyst activity after the introduction of Cu. In the CO_2_-TPD of Figure 4a, it is possible that increased methanol selectivity may also be attributed to the improved surface alkalinity of the bimetallic catalyst [23,39]. In Figure 4b, the introduction of copper is seen to successfully weaken the H_2_ dissociation capability of the cobalt-based catalysts. A comparison of the three catalysts reveals that the peak related to physical adsorption has no obvious change before 150 °C, and the peak related to enhanced chemical adsorption is significantly weakened at 200 °C–600 °C. With the weakening of hydrogen adsorption strength, the free H* species on the catalyst surface decrease during the reaction process, and some adsorbed intermediate species cannot be quickly hydrogenated to CH_4_ in CO_2_ hydrogenation. The chemisorption analysis results suggest that the degradation in the catalyst’s ability to adsorb hydrogen and the changes in the surface alkaline site are some of the factors responsible for the changes in the selectivity of the CO_2_ hydrogenation reaction.

### 3.4. The Catalytic Properties

**Figure 5:**
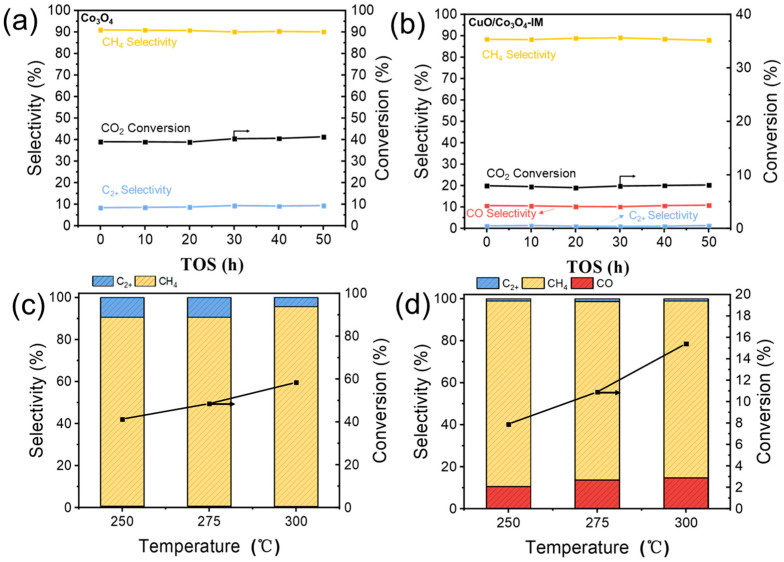
Stability of (**a**) Co_3_O_4_; (**b**) CuO/Co_3_O_4_-IM catalyst at 250 °C. Effect of temperature on CO_2_ conversion and product selectivity of (**c**) Co_3_O_4_; (**d**) CuO/Co_3_O_4_-IM catalyst. Reaction conditions H_2_/CO_2_ = 3/1, p = 2.0 MPa, and GHSV = 6 L·g_cat_^−1^·h^−1^.

Figure 5 explores the stability of the CO_2_ hydrogenation reaction with catalysts Co_3_O_4_ and CuO/Co_3_O_4_-IM and the effect of temperature on the reaction. During the 50 h stability test, the Co_3_O_4_ catalyst exhibited remarkable catalytic stability, as depicted in Figure 5a. Meanwhile, Figure 5b illustrates a substantial rise in CO_2_ conversion rates with increasing reaction temperatures, with product selectivity remaining relatively constant. The CuO/Co_3_O_4_-IM catalyst also demonstrated impressive catalytic stability, as depicted in Figure 5c. Figure 5d indicates that, with an increasing reaction temperature, there is a notable enhancement in CO_2_ conversion, accompanied by some changes in product selectivity, primarily reflected in increased CO selectivity due to thermodynamic effects. At higher temperatures, methanol is absent in the product.

**Figure 6:**
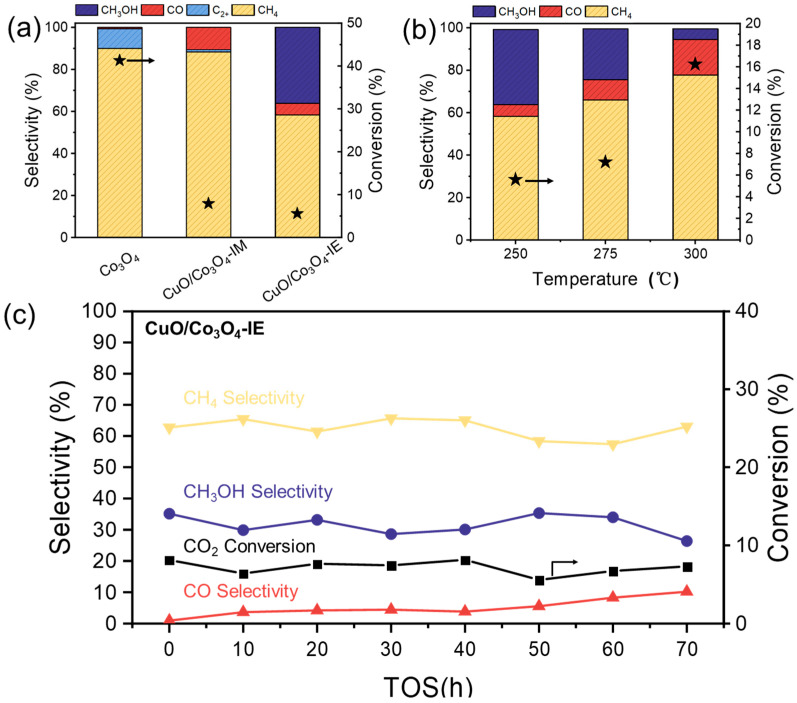
(**a**) Catalytic performance of CO_2_ hydrogenation over CuO/Co_3_O_4_-IE, CuO/Co_3_O_4_-IM, and Co_3_O_4_ catalysts at 250 °C; (**b**) Effect of temperature on the properties of the CuO/Co_3_O_4_-IE catalyst; (**c**) Stability of the CuO/Co_3_O_4_-IE catalyst at 250 °C. Reaction conditions: H_2_/CO_2_ = 3/1; p = 2.0 MPa; GHSV = 6 L·g_cat_^−1^·h^−1^.

The catalytic performance of the three catalysts for the CO_2_ hydrogenation reaction was evaluated (Figure 6a), and the data of the reaction conversion and selectivity are shown in Table 2. The catalytic activity of the Co_3_O_4_ catalyst at 250 °C is considerably higher than that of the other two catalysts containing copper, showing excellent methanation activity. The catalyst prepared using the ion exchange method is seen to produce methanol during the reaction; moreover, due to the special interface between Co and Cu, the synergistic effect between the two can regulate the selectivity of the reaction, reaching a selectivity of 36.1% of methanol. Among the reported Co-Cu catalysts for CO_2_ hydrogenation to methanol, a notably high methanol yield was achieved (Table S6). Through the analysis of the chemisorption and XPS results, it is apparent that the electronic effect of oxygen vacancies on the catalyst surface and the adsorption state of CO_2_ and H_2_ are the primary influencers of product selectivity [27]. Figure 6b,c depicts the temperature-dependent performance and stability test for 70 h of the CuO/Co_3_O_4_-IE catalyst, revealing its relative stability throughout the reaction process. During the 70 h test, both the CO_2_ conversion and methanol selectivity remained unchanged. However, with a rising temperature, there was a substantial increase in CO selectivity due to thermodynamic factors. There are two primary factors contributing to this enhanced methane selectivity: thermodynamics and the cobalt phase transition during CO_2_ hydrogenation (Figure S4). Simultaneously, there was a notable decrease in methanol selectivity. The chromatogram of the CuO/Co_3_O_4_-IE catalytic test is depicted in Figures S5 and S6.

The comparison of the three catalysts demonstrates that the single-component cobalt catalyst still shows excellent methanation capability under conventional conditions and exhibits a clear change in selectivity upon the addition of copper. The difference in the selectivity of the CuO/Co_3_O_4_-IM and CuO/Co_3_O_4_-IE catalysts is attributed to the difference in the method of copper introduction. Due to the excellent RWGS activity of the CuO/Co_3_O_4_-IM catalyst prepared through impregnation, CO appeared in the product; however, it did not appear in the product of the Co_3_O_4_ catalyst. The Co-Cu component of the CuO/Co_3_O_4_-IM catalyst has no obvious synergistic effect, and the two components play their respective independent roles, with cobalt being responsible for the methanation reaction and copper for the RWGS. In the CuO/Co_3_O_4_-IM catalyst, the presence of Co predominantly facilitates methane production while Cu favors CO generation. There is not a synergistic effect between them that leads to the formation of other products. Conversely, in the CuO/Co_3_O_4_-IM catalyst, there exists a discernible interaction between Co and Cu. An electronic effect manifests on the catalysts’ surface, influencing the adsorption of reactants and intermediates. The synergy between these elements prompts the production of methanol during the reaction.

## 4. Conclusions

In this work, we loaded copper onto Co_3_O_4_ nanosheets through ion exchange inverse loading, and the close contact between copper and Co_3_O_4_ nanosheets induced certain interactions to form a special Co-Cu interface, which significantly changes the product selectivity of the cobalt-based catalysts. STEM and SEM results show that the ion exchange method can disperse the copper more evenly across the Co_3_O_4_ nanosheets. A reduction in the temperature program and the XPS results indicate that the metal–metal interaction between Co-Cu is facilitated by the catalyst prepared through the ion exchange method, and the electron transfer from surface defect oxygen restricts the adsorption of CO_2_ and the activation of H_2_ at the catalyst surface, resulting in selective changes. The temperature program desorption of CO_2_ and H_2_ by the three catalysts shows that the close interaction between Co-Cu changes the adsorption of CO_2_, leading to a difference in activity. Moreover, the methanation of CO_2_ is effectively suppressed, and methanol formation is promoted by changes in the alkalinity of the catalyst surface and the weakening of the adsorption ability of hydrogen dissociation. In the performance evaluation of the CO_2_ hydrogenation reaction, the CuO/Co_3_O_4_-IE catalyst exhibits the best reaction performance with a methanol selectivity of 36.1%. This study has constructed a special Co-Cu interface by tuning the interaction between cobalt and copper, which breaks the methanation process of cobalt-based catalysts during CO_2_ hydrogenation and provides a different idea for changing the selectivity of catalytic reactions.

## Figures and Tables

**Table 1:** Chemical compositions of different samples determined using ICP-MS characterizations.

Catalysts	Co (wt%)	Cu (wt%)	Co:Cu (mol/mol)	O (wt%) ^[a]^
1CuO/4Co_3_O_4_-IM	46.0	11.6	3.7	42.4%
1CuO/4Co_3_O_4_-IE	54.5	17.9	3.0	27.6%

^[a]^ The weight percent of oxygen is calculated by subtracting the mass fractions of other components from 100.

**Table 2:** Catalytic performance of CuO/Co_3_O_4_-IE, CuO/Co_3_O_4_-IM, and Co_3_O_4_ catalysts in CO_2_ hydrogenation. Reaction conditions: H_2_/CO_2_ = 3/1; p = 2.0 MPa; GHSV = 6 L·g_cat_^−1^·h^−1^.

Catalysts	X_CO2_ (%)	Selectivity (%)			
		CO	CH_4_	C_2_-C_4_	CH_3_OH
Co_3_O_4_	41.3	0.7	90.0	9.3	~
1CuO/_4_Co3O_4_-IM	7.9	10.6	88.3	1.1	~
1CuO/_4_Co3O_4_-IE	5.6	5.5	58.4	0.8	35.3

## Data Availability

Data available on request.

## Supplementary Materials

The following supporting information can be downloaded at: https://www.mdpi.com/article/10.3390/nano13243153/s1, Figure S1: SEM image of Co(OH)_2_; Figure S2: (a) STEM image of CuO/Co_3_O_4_-IM; (b) EDX elemental mappings of CuO/Co_3_O_4_-IM yellow represents Co, and purple represents Cu; Figure S3: (a) STEM image of CuO/Co_3_O_4_-IE; (b) EDX elemental mappings of CuO/Co_3_O_4_-IE yellow represents Co, and purple represents Cu; Figure S4: XRD patterns of CuO/Co_3_O_4_-IE spent catalysts; Figure S5: Agilent GC 7890B chromatogram of gas phase products of CO_2_ hydrogenation catalyzed by CuO/Co_3_O_4_-IE; Figure S6: Agilent GC 4890D chromatogram of liquid phase products of CO_2_ Hydrogenation catalyzed by CuO/Co_3_O_4_-IE; Table S1: The results of XPS Co 2p_3/2_ for CuO/Co_3_O_4_-IE, CuO/Co_3_O_4_-IM, and Co_3_O_4_ catalysts; Table S2: XPS O 1s fitting peak data of the different catalysts; Table S3: The results of XPS O 1s for CuO/Co_3_O_4_-IE, CuO/Co_3_O_4_-IM, and Co_3_O_4_ catalysts; Table S4: X-ray photoelectron spectroscopy surface elemental concentrations (at.%) as well as the relative surface concentration of the elements; Table S5: Chemisorption amounts of CO and H_2_ over the CuO/Co_3_O_4_-IE, CuO/Co_3_O_4_-IM, and Co_3_O_4_ catalysts; Table S6: State-of-the-art Co-Cu catalysts for methanol synthesis using CO_2_ hydrogenation. Ref. [40] is cited in Supplementary Materials.
