# Supplementary material for: Sheet-Like Morphology CuO/Co3O4 Nanocomposites for Enhanced Catalysis in Hydrogenation of CO2 to Methanol
Source: Nanomaterials (Basel). 2023 Dec 16;13(24):3153. doi: 10.3390/nano13243153 (PMC10745419; doi:10.3390/nano13243153)
Supplement: Supplementary file 1 [file nanomaterials-13-03153-s001.zip › nanomaterials-2739385-supplementary.pdf]

Sheet-like Morphology CuO/Co_3O_4 Nanocomposites for Enhanced Catalysis in Hydrogenation of CO_2 to Methanol

Zhenteng Sheng, Hui Zhou, Yuhua Zhang, Jinlin Li * and Li Wang *

Key Laboratory of Catalysis and Energy Materials Chemistry of Ministry of Education , Hubei Key Laboratory of Catalysis and Materials Science, South-Central Minzu University, Wuhan 430074, China
* Correspondence: lij@mail.scuec.edu.cn (J.L.); li.wang@scuec.edu.cn (L.W.)

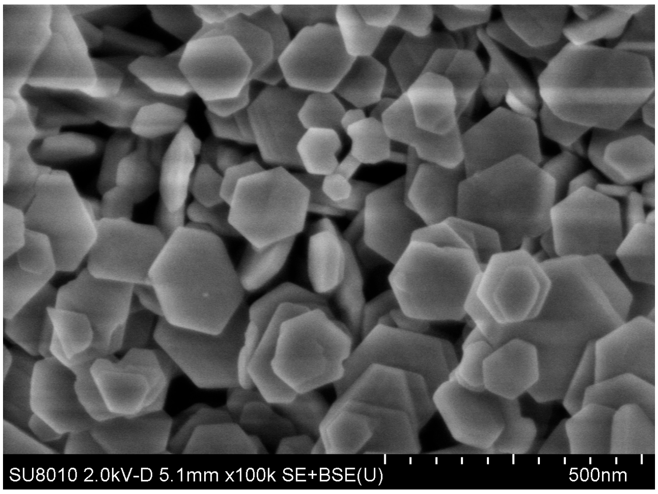

Figure S1. SEM image of $Co(OH)_2$.

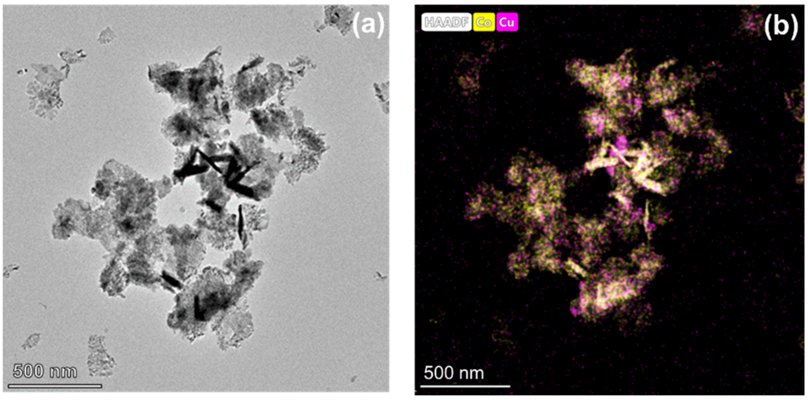

Figure S2. (a) STEM image of CuO/Co_3O_4-IM; (b) EDX elemental mappings of CuO/Co_3O_4-IM yellow represents Co, and purple represents Cu.

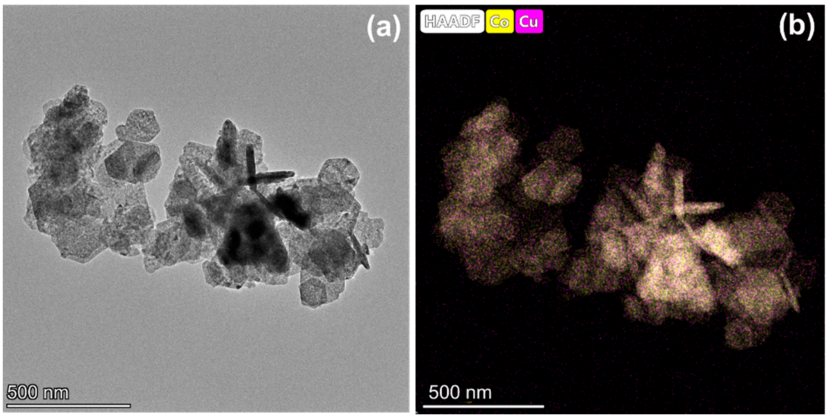

Figure S3. (a) STEM image of CuO/Co_3O_4-IE; (b) EDX elemental mappings of CuO/Co_3O_4-IE yellow represents Co, and purple represents Cu.

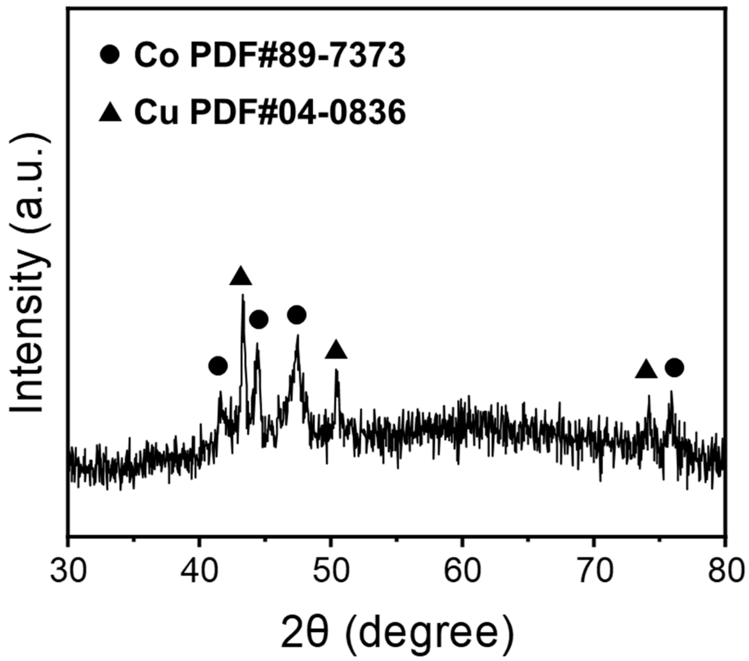

Figure S4. XRD patterns of CuO/Co_3O_4-IE spent catalyst.

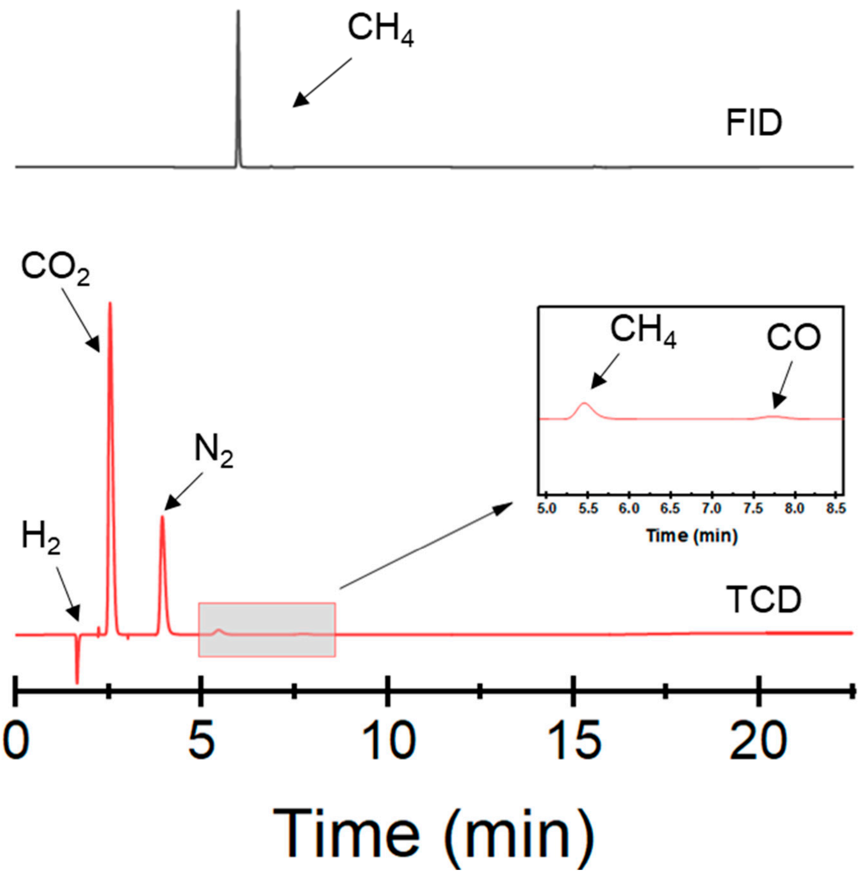

Figure S5. Agilent GC 7890B chromatogram of gas phase products of CO_2 Hydrogenation catalyzed by CuO/Co_3O_4-IE.

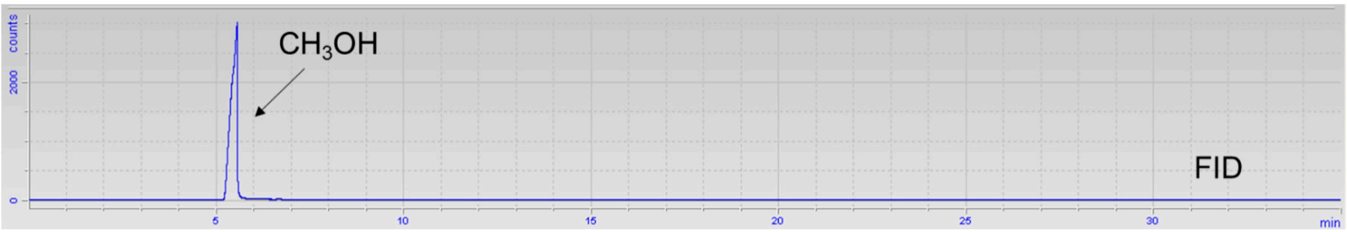

Figure S6. Agilent GC 4890D chromatogram of liquid phase products of CO_2 Hydrogenation catalyzed by CuO/Co_3O_4-IE.

Table S1. The results of XPS Co $2p_{3/2}$ for CuO/Co_3O_4-IE, CuO/Co_3O_4-IM and Co_3O_4 catalysts.

Catalysts	Co $2p_{3/2}$ Binding Energy (eV)	
	Co^{3+}	Co^{2+}
Co_3O_4	779.9	781.5
$1CuO/4Co_3O_4$-IM	779.5	780.9
$1CuO/4Co_3O_4$-IE	779.4	780.8

Table S2. XPS O 1s fitting peak data of the different catalysts.

Catalysts	O (%)	O_c/O_T[a]	O_d /O_T[a]	O_l/O_T[a]
Co_3O_4	71.1%	8.9%	36.6%	54.5%
$1CuO/4Co_3O_4$-IM	66.8%	11.2%	29.5%	59.1%
$1CuO/4Co_3O_4$-IE	65.3%	5.6%	31.0%	63.4%

Table S3. The results of XPS O 1s for CuO/Co_3O_4-IE, CuO/Co_3O_4-IM and Co_3O_4 catalysts.

Catalysts	O 1s Binding Energy (eV)		
	$O_{lattice}$	O_{defect}	$O_{chemisored}$
Co_3O_4	530.0	531.3	533.1
$1CuO/4Co_3O_4$-IM	529.8	531.5	533.2
$1CuO/4Co_3O_4$-IE	529.8	531.8	533.6

Table S4. X-ray photoelectron spectroscopy surface elemental concentrations (at.%) as well as the relative surface concentration of the elements.

Catalysts	Co%	Cu%	O%
Co_3O_4	70.5	0	29.5
$1CuO/4Co_3O_4$-IM	65.3	11.7	23.0
$1CuO/4Co_3O_4$-IE	65.0	6.3	28.7

Table S5. Chemisorption amounts of CO and H_2 over CuO/Co_3O_4-IE, CuO/Co_3O_4-IM and Co_3O_4 catalysts.

Catalysts	CO_2-TPD	H_2-TPD
	CO_2 uptake (μmmol·g_{cat}^{-1})	H_2 uptake (μmmol·g_{cat}^{-1})
Co_3O_4	501.1	374.2
1CuO/4Co_3O_4-IM	450.3	302.1
1CuO/4Co_3O_4-IE	393.7	258.6

Table S6. State of art Co-Cu catalysts for methanol synthesis by CO_2 hydrogenation. .

Catalysts	T (℃)	p (MPa)	MeOH Selectivity(%)	MeOH STY
5Cu5CoZ[1]	190	3	76.1	0.62 mmol·g_{gat}^{-1}·h^{-1}
1CuO/4Co_3O_4-IE	250	2	36.1	1.23 mmol·g_{gat}^{-1}·h^{-1}

1. Din, I.U.; Alotaibi, M.A.; Alharthi, A.I.; Al-Shalwi, M.N.; Alshehri, F. Green Synthesis Approach for Preparing Zeolite Based Co-Cu Bimetallic Catalysts for Low Temperature CO_2 Hydrogenation to Methanol. *Fuel* **2022**, *330*, 125643.
